# KPC-2 β-lactamase enables carbapenem antibiotic resistance through fast deacylation of the covalent intermediate

**DOI:** 10.1074/jbc.RA120.015050

**Published:** 2020-12-10

**Authors:** Shrenik C. Mehta, Ian M. Furey, Orville A. Pemberton, David M. Boragine, Yu Chen, Timothy Palzkill

**Affiliations:** 1 Department of Pharmacology and Chemical Biology, Baylor College of Medicine, Houston, Texas, USA; 2 Department of Molecular Medicine, University of South Florida Morsani College of Medicine, Tampa, Florida, USA; 3 Verna and Marrs McLean Department of Biochemistry and Molecular Biology, Baylor College of Medicine, Houston, Texas, USA

**Keywords:** antibiotic resistance, enzyme catalysis, enzyme kinetics, protein structure, microbiology, enzyme mechanism, PDB, Protein Data Bank

## Abstract

Serine active-site β-lactamases hydrolyze β-lactam antibiotics through the formation of a covalent acyl-enzyme intermediate followed by deacylation *via* an activated water molecule. Carbapenem antibiotics are poorly hydrolyzed by most β-lactamases owing to slow hydrolysis of the acyl-enzyme intermediate. However, the emergence of the KPC-2 carbapenemase has resulted in widespread resistance to these drugs, suggesting it operates more efficiently. Here, we investigated the unusual features of KPC-2 that enable this resistance. We show that KPC-2 has a 20,000-fold increased deacylation rate compared with the common TEM-1 β-lactamase. Furthermore, kinetic analysis of active site alanine mutants indicates that carbapenem hydrolysis is a concerted effort involving multiple residues. Substitution of Asn170 greatly decreases the deacylation rate, but this residue is conserved in both KPC-2 and non-carbapenemase β-lactamases, suggesting it promotes carbapenem hydrolysis only in the context of KPC-2. X-ray structure determination of the N170A enzyme in complex with hydrolyzed imipenem suggests Asn170 may prevent the inactivation of the deacylating water by the 6α-hydroxyethyl substituent of carbapenems. In addition, the Thr235 residue, which interacts with the C3 carboxylate of carbapenems, also contributes strongly to the deacylation reaction. In contrast, mutation of the Arg220 and Thr237 residues decreases the acylation rate and, paradoxically, improves binding affinity for carbapenems. Thus, the role of these residues may be ground state destabilization of the enzyme-substrate complex or, alternatively, to ensure proper alignment of the substrate with key catalytic residues to facilitate acylation. These findings suggest modifications of the carbapenem scaffold to avoid hydrolysis by KPC-2 β-lactamase.

The carbapenem class of β-lactam antibiotics are effective in treating severe infections caused by Gram-negative bacterial pathogens such as *Escherichia coli*, *Klebsiella pneumoniae*, and *Pseudomonas aeruginosa* (1). Formerly, the carbapenems were reserved as the last line of therapy and were used upon failure of other treatment options (1). However, with increased resistance to other β-lactam classes such as penicillins and cephalosporins, the frequency of carbapenem administration for treating clinical infections is increasing (2).

β-Lactam antibiotics, including carbapenems, act by binding and inactivating a group of essential enzymes, the penicillin binding proteins, resulting in inhibition of peptidoglycan synthesis and ultimately cell death (3). Carbapenems are characterized by a 4:5 fused lactam ring system with an unsaturated C2-C3 bond (Fig. 1) (1). However, the most distinguishing feature of this class is the 6α-1R-hydroxyethyl side chain at position C6 (Fig. 1) (4). Conventional β-lactam antibiotics such as penicillins and cephalosporins have a 6β-acylamide substituent at this position (Fig. 1). This change in stereochemistry at the C6 position is important for the potency and broad-spectrum activity of the carbapenem antibiotics (5, 6, 7).

**Figure 1:**
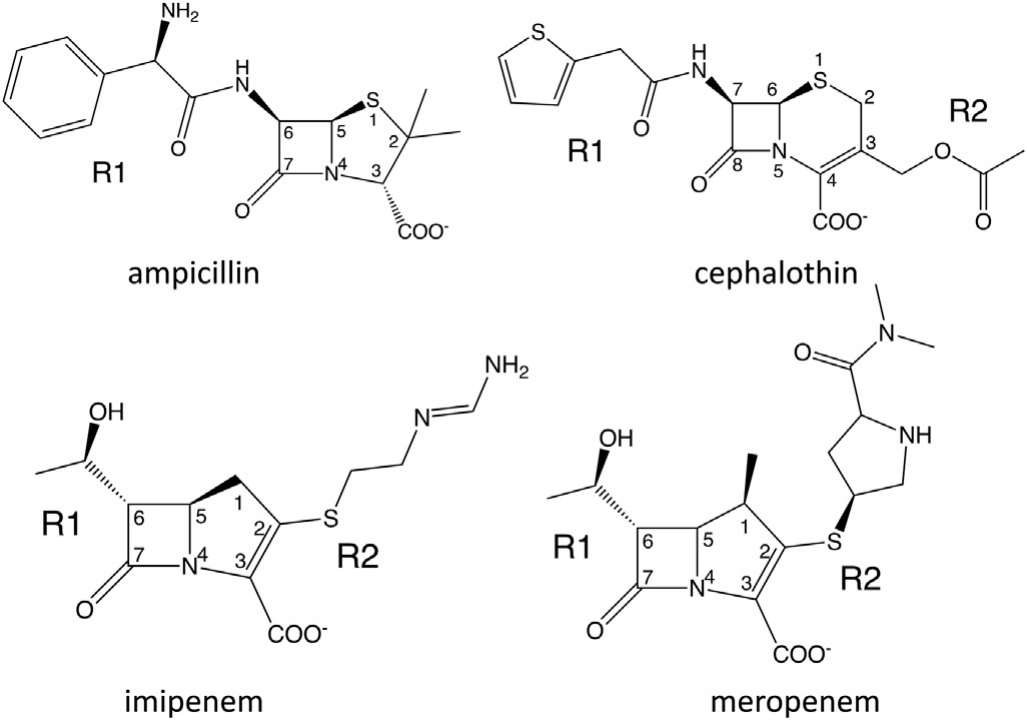
**β-Lactam antibiotics used in this study.** The R1 and R2 groups represent chemical substituents that vary within the class. Key atom positions are numbered.

One of the most important characteristics of carbapenems is that they are poorly hydrolyzed by most β-lactamases (1). The most common mechanism of resistance to β-lactam antibiotics in Gram-negative bacteria is the production of β-lactamases. There are four classes of β-lactamases (A, B, C, D) based on the primary amino acid sequence homology (8). Classes A, C, and D are serine hydrolases, whereas class B consists of zinc metalloenzymes. Class B enzymes, including the New Delhi metallo-β-lactamase, can efficiently hydrolyze carbapenems, whereas most of the serine hydrolases do not (9). In fact, carbapenems inhibit many clinically important class A β-lactamase enzymes such as TEM-1, SHV-1, and *Mycobacterium tuberculosis* BlaC through formation of a stable, long-lived, acyl-enzyme complex (5, 6, 10, 11).

Several serine β-lactamases belonging to classes A and D, however, have emerged that can hydrolyze carbapenems. Among the class A β-lactamases, several enzymes including KPC, SFC, NMC, IMI, SME that possess carbapenemase activity have been identified in environmental and pathogenic organisms (12, 13, 14, 15, 16, 17, 18). In particular, KPC-producing organisms are now widespread worldwide (19, 20, 21, 22, 23).

Class A β-lactamases, including KPC-2, hydrolyze the amide bond in β-lactam antibiotics through sequential acylation and deacylation steps (24) (Fig. 2). The catalytic Ser70 residue attacks the carbonyl carbon of the β-lactam resulting in cleavage of the amide bond and formation of a covalent acyl-enzyme intermediate. Subsequently, Glu166 activates a catalytic water molecule for attack on the carbonyl carbon of the acyl-enzyme leading to hydrolysis of the ester bond between Ser70 and the drug. Deacylation regenerates the active enzyme and releases the inactivated drug (24). These reactions can be represented in a minimal kinetic scheme as shown in Figure 2.

**Figure 2:**
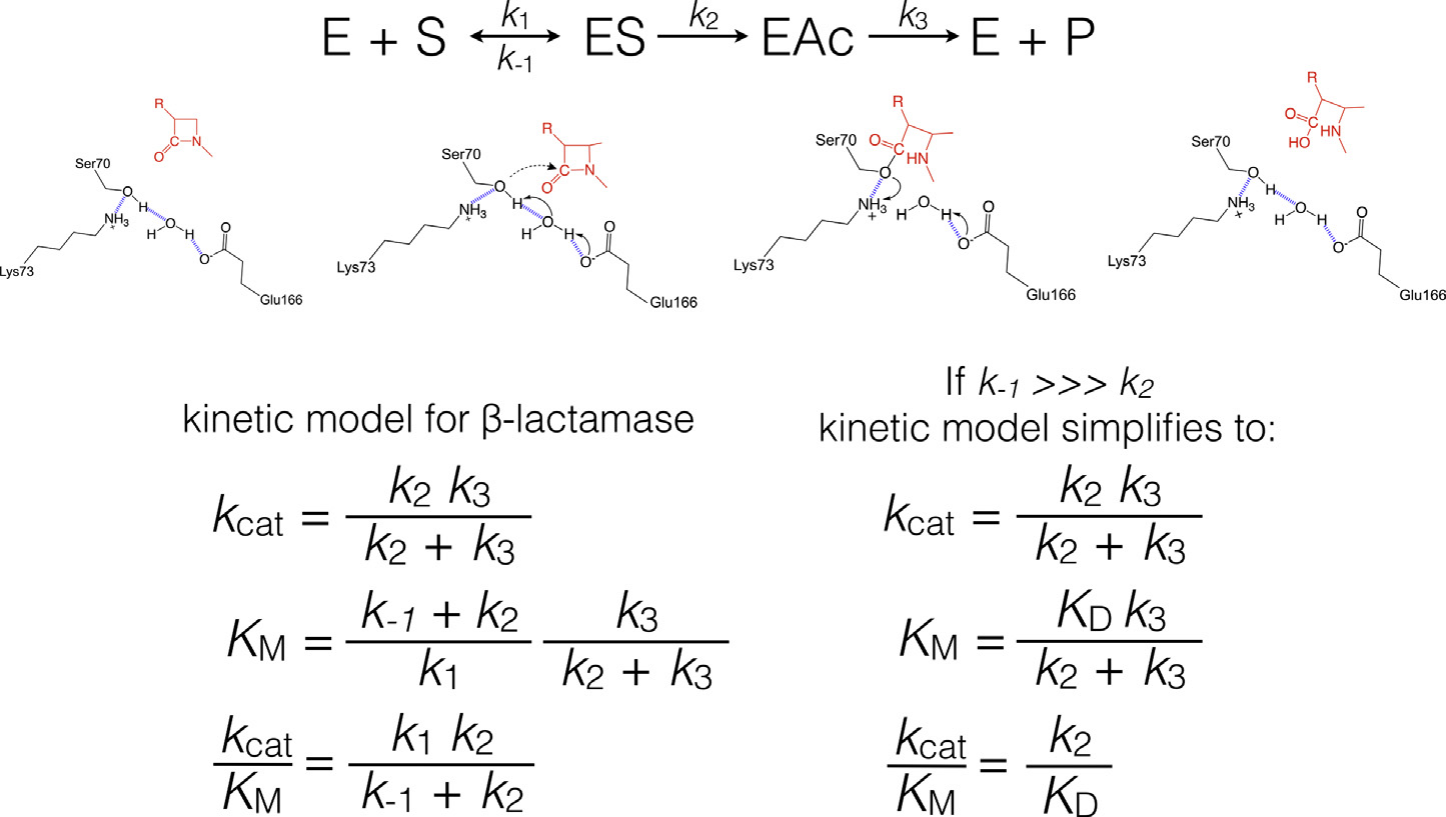
**Kinetic model for serine active site β-lactamases.** At the top is the minimal scheme for the reaction with E as free enzyme, S as substrate, ES as enzyme–substrate complex, EAc as acyl-enzyme intermediate, and P as product. The middle panel shows a schematic of the β-lactamase mechanism with Ser70 serving as the nucleophile for attack on the β-lactam carbonyl carbon to form the acyl-enzyme intermediate. Subsequently, Glu166 acts as a base to activate water for attack on the carbonyl carbon of the acyl-enzyme intermediate leading to hydrolyzed product. At the bottom are the equations for *k*_cat_, *K*_M_, and *k*_cat_/*K*_M_ based on the kinetic scheme shown at the top. A simplified model is shown at right based on the assumption that *k*_−1_>>>*k*_2_.

Structural and biochemical studies on the reaction of imipenem and meropenem with the TEM-1 and SHV-1 enzymes have provided insights into the mechanism of β-lactamase inhibition by carbapenems (5, 6). Nucleophilic attack by the catalytic serine, Ser70, on the β-lactam results in a carbapenem acyl-enzyme complex that is stable owing to a very slow deacylation reaction (Figs. 2 and 3). A number of studies, including X-ray and Raman crystallography and computational simulations have implicated the following factors in stabilization of the acyl-enzyme intermediate: (1) decreased nucleophilicity of the water molecule responsible for deacylation because of a hydrogen bond formed with the 6α-1R-hydroxyethyl group of the carbapenem (7); (2) tautomerization of the acyl-enzyme intermediate from the Δ^2^ to Δ^1^ pyrroline form, which is even more slowly deacylated (10, 25) (Fig. 3); (3) disruption of the deacylation transition state due to displacement of the acyl-enzyme carbonyl oxygen from the oxyanion hole (formed by the backbone –NH of Ser70 and Thr237) (5, 6, 26). For efficient deacylation, it is essential that all the above-mentioned inhibitory traps be avoided. Failure to avoid these traps, as in the case of TEM-1 and SHV-1, leads to inhibition that is characterized by a very slow deacylation reaction and biphasic, branched kinetic progress curves (27).

**Figure 3:**
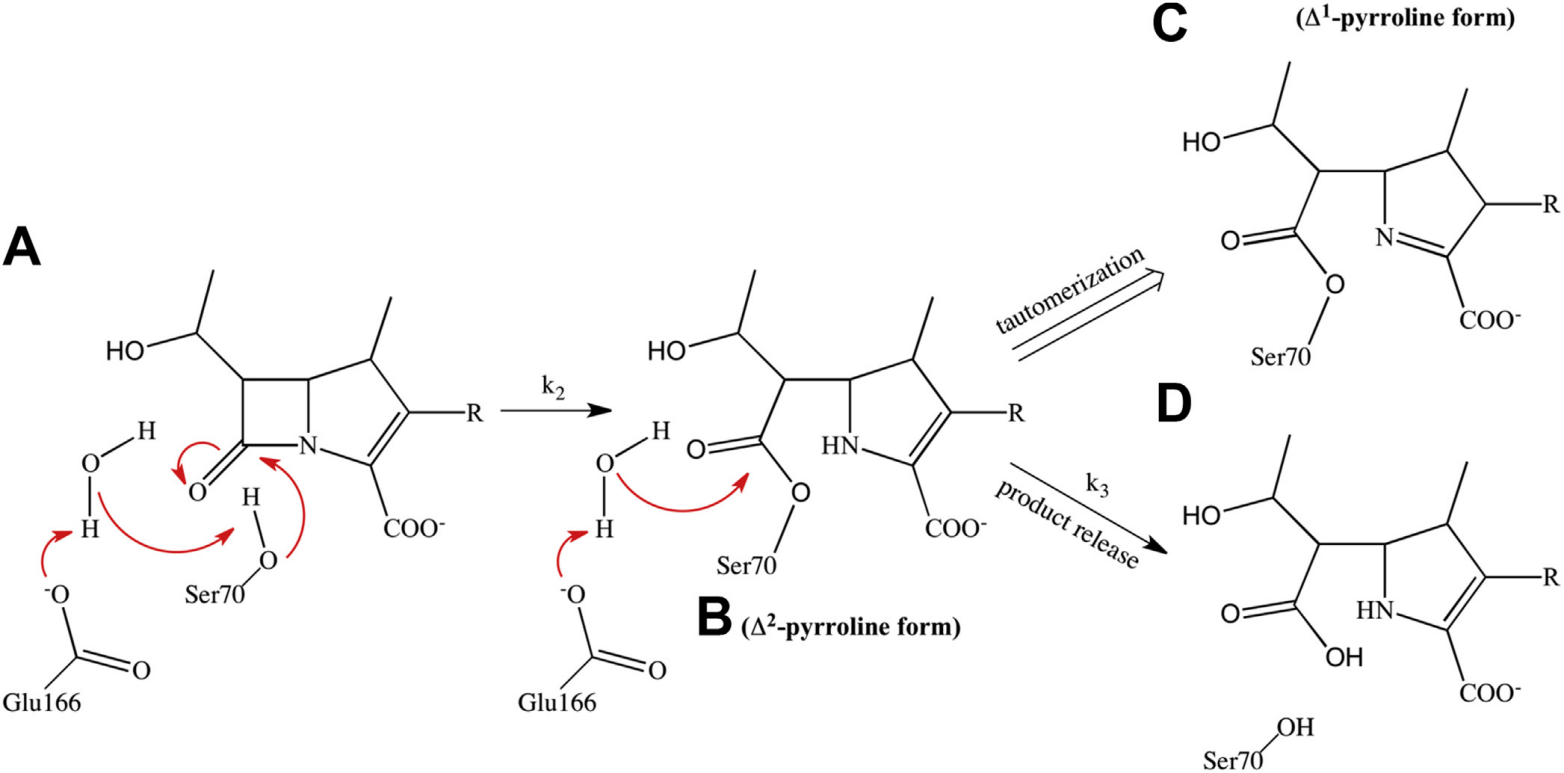
**Mechanism of carbapenem hydrolysis.***A*, activation of Ser70 for nucleophilic attack on the β-lactam carbonyl carbon *via* Glu166 acting through a water molecule. Note that activation through Lys73 acting as the general base is also possible. *B*, activation of a water molecule for attack on the carbonyl carbon of the acyl-enzyme intermediate. *C*, tautomerization of the carbapenem to the Δ^1^-pyrroline form through protonation of C2 and shifting of the double bond to C3-N4. The Δ^1^-pyrroline form deacylates slowly. *D*, deacylation and release of the Δ^2^ pyrroline form.

How then, does KPC-2 avoid the traps discussed above and efficiently deacylate carbapenems? A structural alignment of KPC-2 with the non-carbapenem hydrolyzing TEM-1 β-lactamase reveals an overall broad similarity between the structures with much of the variation due to differences in non–active site loops (Fig. 4*A*). As seen in Figure 4*B*, many of the active site residues are conserved between KPC-2 and TEM-1 and occupy similar positions. Although there are not obvious distinctions to explain the carbapenemase activity of KPC-2 *versus* the lack of activity in TEM-1, subtle alterations in the structure or dynamics of KPC-2 could allow conserved residues to contribute to carbapenem hydrolysis in the context of the KPC-2 enzyme but not in the context of the TEM-1 enzyme (28). For example, the KPC-2 active site is larger and more hydrophobic than non-carbapenemase class A enzymes such as TEM-1, which may result in altered conformations of the hydroxyethyl group that are not accessible to non-carbapenemase enzymes and that do not inactivate the deacylation of water molecule (29, 30). Therefore, we examined the contributions of both conserved and nonconserved active site positions to carbapenem hydrolysis by creating alanine substitutions and characterizing the kinetic properties of the mutated enzymes.

**Figure 4:**
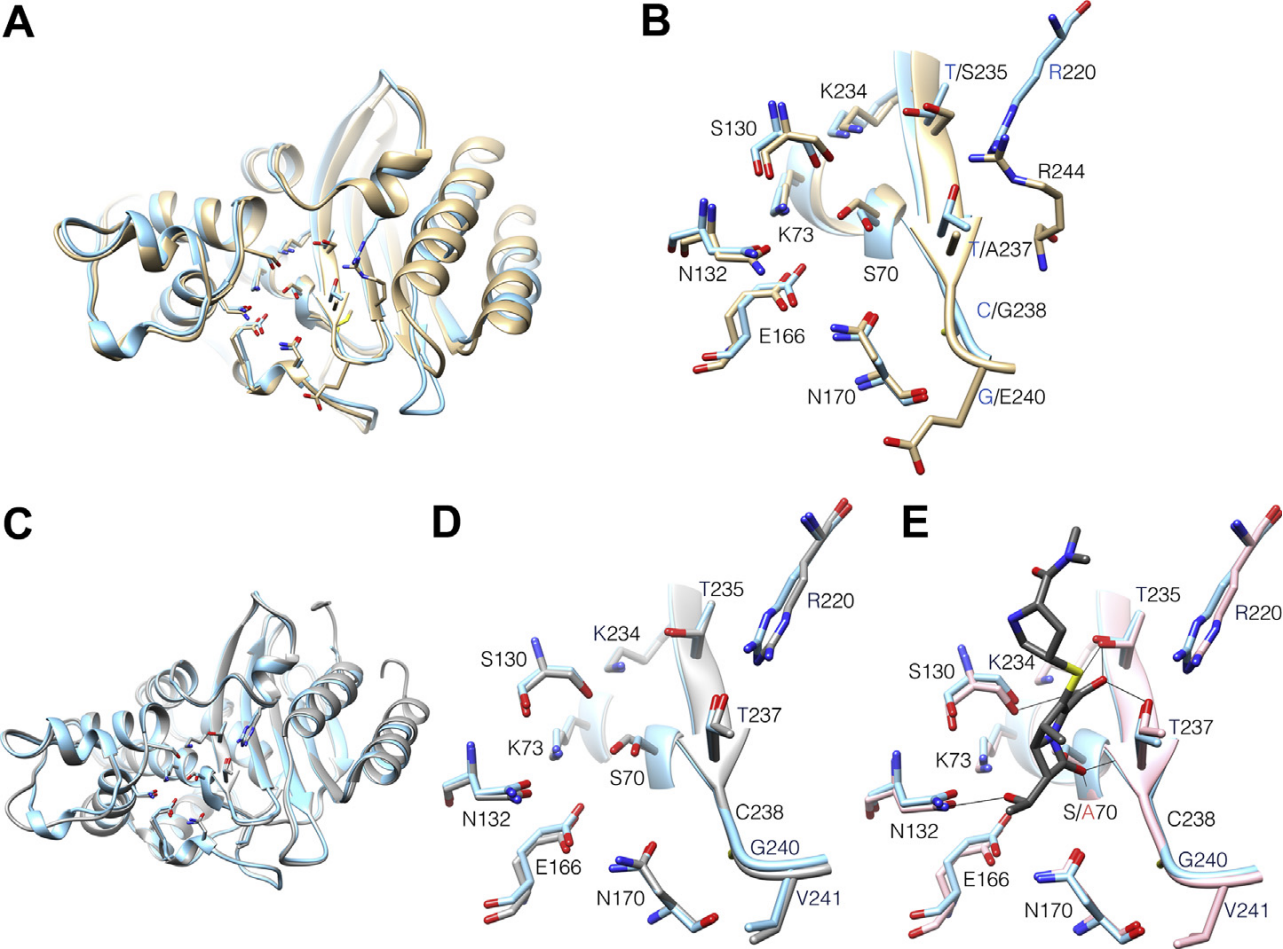
**Structure alignments of KPC-2, TEM-1, and SFC-1 β-lactamases.***A*, ribbon diagram of structural alignment of the non-carbapenemase TEM-1 β-lactamase (*tan*) (PDB ID: 1XPB) with KPC-2 carbapenemase (*light blue*) (PDB ID: 2OV5, chain C). *B*, active site region of structural alignment of TEM-1 (*tan*) and KPC-2 (*light blue*) enzymes from *A*. Active site residues are labeled. Oxygen is shown in *red* and nitrogen in *blue*. *C*, ribbon diagram of the structural alignment of the SFC-1 (*gray*) (PDB ID: 4EQI, chain B) and KPC-2 (*light blue*) (PDB ID: 2OV5, chain C) carbapenemases. *D*, active site region of the structural alignment of SFC-1 (*gray*) and KPC-2 (*light blue*) enzymes from *C*. Active site residues are labeled. Note the conservation of active site residues for SFC-1 *versus* KPC-2. *E*, structural alignment of active site region of KPC-2 (*light blue*) (PDB ID: 2OV5, chain C) with that of SFC-1 S70A mutant (*pink*) in complex with meropenem (*gray*) (PDB ID: 4EUZ). Hydrogen bonds between meropenem and SFC-1 S70A residues are shown as *thin black lines*. Note that the meropenem C3 carboxylate group makes hydrogen bonds to Thr235 and Thr237 and electrostatic interactions with Arg220. Structural equivalents of Thr235 and Arg220 are present in the non-carbapenemase TEM-1 in the form of Ser235 and Arg244 while position 237 is alanine in TEM-1.

Sequence alignments of active-site residues of KPC-2 with other carbapenem-hydrolyzing and non-carbapenem-hydrolyzing β-lactamases reveal that Glu166 and Asn170 are highly conserved among class A β-lactamases (Table 1) where they play a role in deacylation of the acyl-enzyme complex (24, 31). In addition, analysis of the SFC-1 carbapenemase (69% sequence identity to KPC-2) cocrystal structure in complex with meropenem identified residues Thr235, Thr237, and Arg220, which are not conserved in TEM-1, to play a role in binding of the substrate (7) (Fig. 4, *D*–*E*). In the SFC-1 S70A meropenem structure (S70A mutation prevents hydrolysis), the side chains of residues Thr235 and Thr237 interact with the C3 carboxyl group while the Thr237 main-chain nitrogen forms the oxyanion hole along with that of residue 70 (7). The Arg220 residue forms hydrogen bonds with the Thr237 side chain, and the positive charge associated with the residue may contribute to substrate binding by providing an environment favorable for the negatively charged C3 carboxylate of carbapenems (29, 30). In addition, the structure of KPC-2 in complex with hydrolyzed faropenem identified similar interactions with hydrogen bonds between Thr235 and Thr237 and the C3 carboxylate with Arg220 forming a hydrogen bond with Thr237 (30). In order to examine the role of these residues in carbapenem hydrolysis by KPC-2, we created alanine mutants at positions 166, 170, 220, 235, and 237 and used pre-steady-state kinetic analysis to examine their contribution to the acylation and deacylation rates for turnover of the carbapenem, imipenem. In addition, R220Q was created to test the importance of positive charge at this position. The results indicate that, although the deacylation rate is still slower than acylation, wildtype KPC-2 β-lactamase hydrolyzes carbapenems efficiently because of a deacylation rate that is orders of magnitude faster than that previously observed for TEM-1 (31). In addition, the results show residues Asn170, Arg220, and Thr237 all enhance both the acylation and deacylation rates. Substitution of residue Asn170, however, results in the largest reduction in *k*_cat_ for imipenem hydrolysis due to a >3000-fold decrease in the deacylation rate. Interestingly, although acylation and deacylation rates are not known, the N170A substitution had only modest effects on *k*_cat_ for ampicillin and cephalothin catalysis, suggesting that Asn170 is critical for carbapenem but not penicillin or cephalosporin hydrolysis by KPC-2 β-lactamase.

**Table 1:** Alignment of select active site residues of common class A β-lactamases and carbapenemases

β-lactamase	Position						*k*_cat_ (s^−1^)^b^ (ref)
	166	170	220	235	237	244	
TEM-1	E	N	L	S	A	R	0.003 (31)
SHV-1	E	N	L	T	A	R	ND
SME^a^	E	N	R	T	S	A	185 (13)
NMC-A^a^	E	N	R	T	S	A	130 (15)
SFC-1^a^	E	N	R	T	T	A	54 (18)
KPC-2^a^	E	N	R	T	T	A	44 (this paper)
GES-1	E	G	T	T	T	R	0.006 (32)
GES-2^a^	E	N	T	T	T	R	0.012 (32)
GES-5^a^	E	S	T	T	T	R	0.44 (32)

a Carbapenemase enzymes.

b *k*_cat_ values for imipenem hydrolysis.

## Results and discussion

In order to examine the basis of carbapenemase activity of KPC-2 and the roles of specific active-site residues in enzyme function, purified wildtype KPC-2 and the E166A, N170A, R220A, R220Q, T235A, and T237A mutant enzymes were subjected to steady-state and pre-steady-state kinetic studies. Traditional steady-state enzyme kinetic parameters were obtained for the hydrolysis of ampicillin, a penicillin, cephalothin, a first-generation cephalosporin, and the carbapenems imipenem and meropenem. Studies with other carbapenemases such as GES-2 and GES-5 have revealed that nonbulky carbapenems such as imipenem have different kinetic parameters compared with bulky carbapenems such as meropenem, doripenem, and ertapenem due to different binding modes in the enzyme active site (32) (Fig. 1). Thus, imipenem and meropenem were chosen to represent both nonbulky and bulky carbapenems. Non-carbapenemase class A β-lactamases are inhibited by carbapenems owing to a very slow deacylation rate (5, 6, 27). Therefore, the rates of acylation and deacylation for KPC-2 and the mutants were determined in single turnover pre-steady-state kinetic experiments with imipenem as substrate to examine the rate of acylation and, by inference, the deacylation rate.

### Steady-state and pre-steady-state kinetic parameters of KPC-2 β-lactamase

The wildtype KPC-2 enzyme displayed specificity constants (*k*_cat_/*K*_M_) of 2.3 × 10^5^, 1.0 × 10^6^, 1.6 × 10^5^, and 1.4 × 10^5^ M^−1^ s^−1^ for ampicillin, cephalothin, imipenem, and meropenem hydrolysis, respectively (Table 2) (Figs. S1–S4). Thus, ampicillin, imipenem, and meropenem are hydrolyzed at similar rates, whereas cephalothin is hydrolyzed at an ∼10-fold faster rate. In comparison with the non-carbapenemase TEM-1 enzyme, the specificity constant displayed by KPC-2 for ampicillin hydrolysis is 170-fold lower (33). In contrast, the TEM-1 enzyme essentially does not hydrolyze imipenem and meropenem and so KPC-2-mediated hydrolysis of these substrates is much faster.

**Table 2:** Steady-state kinetic parameters for KPC-2 and mutant β-lactamases

	AMP	CEP	IMI	MERO
KPC-2				
*k*_cat_ (s^−1^)	47 ± 3	142 ± 14	44 ± 1	3.4 ± 0.1
*K*_m_ (μM)	203 ± 35	135 ± 35	275 ± 15	24 ± 1.1
*k*_cat_/*K*_m_ (μM^−1^ s^−1^)	0.23 ± 0.04	1.0 ± 0.2	0.16 ± 0.01	0.14 ± 0.04
E166A				
*k*_cat_ (s^−1^)	HND^a^	HND^a^	HND^a^	HND^a^
*K*_m_ (μM)	HND^a^	HND^a^	HND^a^	HND^a^
*k*_cat_/*K*_m_ (μM^−1^ s^−1^)	HND^a^	HND^a^	HND^a^	HND^a^
N170A				
*k*_cat_ (s^−1^)	12 ± 1	133 ± 9	0.02 ± 0.0006	0.001
*K*_m_ (μM)	55 ± 5	1126 ± 136	≤0.6 ± 0.01	≤12
*k*_cat_/*K*_m_ (μM^−1^ s^−1^)	0.22 ± 0.02	0.12 ± 0.02	≥0.03 ± 0.001	≥0.0001
R220A				
*k*_cat_ (s^−1^)	747 ± 20	427 ± 16	13 ± 0.3	0.30 ± 0.01
*K*_m_ (μM)	451 ± 34	702 ± 57	22 ± 3	≤1.7 ± 0.02
*k*_cat_/*K*_m_ (μM^−1^ s^−1^)	1.7 ± 0.1	0.61 ± 0.06	0.59 ± 0.08	≥0.17 ± 0.01
R220Q				
*k*_cat_ (s^−1^)	497 ± 26	489 ± 14	27 ± 1	0.90 ± 0.02
*K*_m_ (μM)	131 ± 14	378 ± 29	29 ± 2	≤4.7 ± 1.0
*k*_cat_/*K*_m_ (μM^−1^ s^−1^)	3.8 ± 0.4	1.3 ± 0.1	1.0 ± 0.1	≥0.19 ± 0.03
T237A				
*k*_cat_ (s^−1^)	153 ± 4	47 ± 1	8.9 ± 0.3	0.11 ± 0.01
*K*_m_ (μM)	17 ± 2	51 ± 4	19 ± 2	1.3 ± 0.24
*k*_cat_/*K*_m_ (μM^−1^ s^−1^)	9.0 ± 0.1	0.92 ± 0.08	0.47 ± 0.06	0.09 ± 0.02
T235A				
*k*_cat_ (s^−1^)	ND^b^	ND^b^	1.0 ± 0.03	0.27 ± 0.01
*K*_m_ (μM)	>2500	>800	81 ± 7	17 ± 3.4
*k*_cat_/*K*_m_ (μM^−1^ s^−1^)	1.5 ± 0.04	0.05 ± 0.001	0.01 ± 0.001	0.02 ± 0.004

a HND, hydrolysis not detected.

b ND, not determined, high *K*_m_.

**Figure 5:**
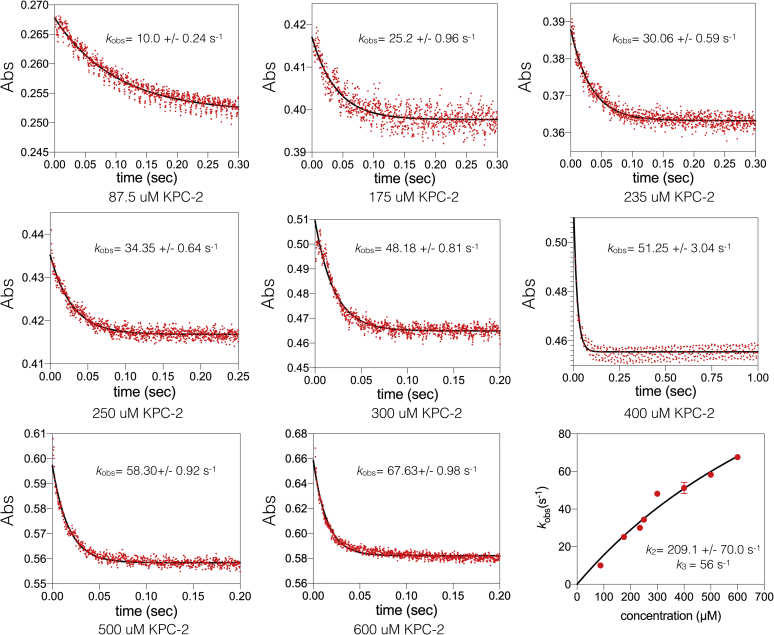
**Single turnover kinetic analysis of wildtype KPC-2 carbapenemase hydrolysis of imipenem.** Imipenem, 10 μM, was used with increasing concentrations of KPC-2 enzyme as indicated below each plot. Absorbance is shown on the y-axis and time in seconds on the x-axis. The *k*_obs_ value obtained from fitting a single exponential equation is indicated for each plot. At the bottom right is the fit of the *k*_obs_ values *versus* the KPC-2 enzyme concentrations to a hyperbola to obtain the acylation rate (*k*_2_).

The minimal kinetic model for β-lactam hydrolysis by serine β-lactamases is shown along with the equations for *k*_cat_, *K*_M,_ and *k*_cat_/*K*_M_ in Figure 2 (32, 34, 35). In the kinetic model, *k*_cat_ is dependent on the magnitude and relationship between *k*_2_ and *k*_3_. Note that the minimal kinetic model does not include a product release step (Fig. 2). Product release is assumed to be fast relative to other steps for β-lactamases, but it is possible the *k*_3_ values could be affected by slow product release. Single turnover kinetics with an excess of KPC-2 over imipenem substrate was used to determine the acylation rate (*k*_2_), whereas the deacylation rate (*k*_3_) was calculated using the experimental values of *k*_2_ and *k*_cat_ by substitution in Equation 5, similar to the approach used for serine proteases and other β-lactamases (36, 37, 38) (Experimental Procedures). The results indicated an acylation rate (*k*_2_) of 209 s^−1^ and a deacylation rate (*k*_3_) of 56 s^−1^ for imipenem hydrolysis (Table 3, Fig. 5). Since the acylation and deacylation rates are broadly similar (within fourfold), KPC-2 does not have a strongly rate-limiting step during imipenem hydrolysis. Non-carbapenemase class A enzymes such as TEM-1 and SHV-1 exhibit very slow deacylation rates for carbapenem turnover (27, 33). The deacylation rate of imipenem by KPC-2 is 20,000-fold higher than that observed for TEM-1 (33). Thus, a fast deacylation rate for KPC-2 as compared with TEM-1 allows the successful turnover of carbapenem substrates.

**Table 3:** Acylation and deacylation rates for imipenem hydrolysis

β-lactamase	*k*_2_ (s^−1^)^a^	*k*_3_ (s^−1^)^a^	*K*_D_ (μM)^a^
KPC-2	209 ± 70	56 ± 20^b^	1243
N170A	40 ± 13	0.02 ± 0.007	220 ± 51
R220A	35 ± 6	20.7 ± 3.9	188 ± 6.3
R220Q	34 ± 3	131 ± 18	35 ± 9.4
T237A	18 ± 1	17.6 ± 1.6	24 ± 3.4

a *k*_2_ and *K*_D_ were determined based on fitting single turnover results (*k*_obs_) to the equation *k*_obs_ = (*k*_2_∗E)/(*K*_D_ + E). *k*_3_ was calculated from *k*_2_ and *k*_cat_ using the equation *k*_cat_ = (*k*_2_∗*k*_3_)/(*k*_2_ + *k*_3_).

b The error on *k*_3_ was determined by propagating the error on the *k*_2_ and *k*_cat_ determinations by adding the proportional error from each and multiplying that value by the *k*_3_ value.

It was also of interest to examine the binding affinity of KPC-2 for imipenem. The fit of the *k*_obs_ values to obtain *k*_2_ also yielded a *K*_D_ value of 1240 μM, which is approximately fivefold higher than the *K*_M_ (Tables 2 and 3). In the β-lactamase kinetic scheme the relationship between *K*_M_ and the binding affinity for substrate (dissociation constant *K*_D_) depends not only on the affinity but also on the relative values of *k*_2_ and *k*_3_ (Fig. 2) (35). Based on the assumption that *k*_−1_ >> *k*_2_, *k*_cat_/*K*_M_ = *k*_2_/*K*_D_ and thus *K*_D_ was also estimated based on experimentally determined *k*_cat_/*K*_M_ and *k*_2_ values to yield a *K*_D_ value (Table 3). A relatively weak binding affinity for the substrate is consistent with a rapid acylation rate because very tight ground state binding requires a higher energy to reach the transition state, thereby resulting in slower reaction rates (39).

A detailed analysis of the kinetics of carbapenem hydrolysis has previously been performed for the class A GES-1, -2, and -5 enzymes (36). These enzymes differ from one another by the residue at position 170, with GES-1 containing Gly170 and GES-2 and GES-5 containing Asn170 and Ser170, respectively. The GES-2 and GES-5 enzymes are carbapenemases with GES-5 exhibiting the highest carbapenem hydrolysis levels (36). The specificity constants for imipenem hydrolysis by GES-5 and KPC-2 are similar with values of ∼2 to 3 × 10^5^ M^−1^ s^−1^ (Table 2) (32). There are large differences, however, in the kinetic parameters *k*_cat_ and *K*_M_ as well as *k*_2_ and *k*_3_ for imipenem hydrolysis by KPC-2 *versus* GES-5. *k*_cat_ and *K*_M_ values are 100- and 200-fold higher, respectively, for KPC-2, whereas the acylation rate is 6-fold higher and the deacylation rate is 120-fold higher for KPC-2 *versus* GES-5 (36) (Tables 2 and 3). There is also a 2500-fold difference in affinity for the imipenem substrate with *K*_D_ values of 1240 μM for KPC-2 and 0.5 μM for GES-5 (36) (Table 3). Thus, while GES-5 binds imipenem tightly, KPC-2 exhibits a much faster turnover due to rapid acylation and deacylation rates. These results indicate that at low concentrations of substrate (<<*K*_M_) GES-5 and KPC-2 have similar carbapenemase activity, but at saturating substrate (S >> *K*_M_), KPC-2 is more effective at carbapenem hydrolysis.

### Role of Glu166 and Asn170 in hydrolysis of β-lactam antibiotics

Glu166 has been shown to be required for the deacylation reaction in class A β-lactamases (24, 40) (Fig. 4). X-ray structures of class A enzymes show that the Glu166 side chain coordinates and activates a water molecule for deacylation of the acyl-enzyme species (6, 7, 29, 40, 41, 42) (Fig. 2). In addition, crystal structures of trapped acyl-enzymes have been observed for Glu166 mutants of several class A enzymes (5, 6, 7, 40, 43). Therefore, we anticipated that replacing Glu166 with alanine would result in loss of enzymatic activity due to the lack of an activated hydrolytic water molecule. Consistent with this hypothesis, steady-state kinetic parameters for the KPC-2 E166A mutant could not be obtained for ampicillin, imipenem, and meropenem as no measurable substrate hydrolysis was observed up to an enzyme concentration of 10 μM. Cephalothin turnover was observed with 10 μM of the E166A enzyme; however, the kinetic parameters were not calculated. Thus, consistent with previous observations, the Glu166 residue of KPC-2 plays an essential role in the hydrolysis of β-lactam antibiotics and mutating this residue largely eliminates enzyme activity.

Asn170 is conserved among nearly all class A β-lactamases (Table 1) (Fig. 4), including KPC-2. Asn170 along with Glu166 coordinates the hydrolytic water molecule critical for the deacylation reaction. In the non-carbapenemase TEM-1 β-lactamase, replacing Asn170 with Ala results in a 17-fold decrease in the specificity constant for ampicillin hydrolysis (44). Therefore, the Asn170 residue contributes but is not absolutely required for ampicillin hydrolysis by TEM-1. Thus, we hypothesized that the N170A mutation in KPC-2 would decrease but not abolish hydrolysis of β-lactam substrates. Surprisingly, the specificity constant of KPC-2 N170A for ampicillin hydrolysis remained unchanged as compared with wildtype KPC-2, although both *k*_cat_ and *K*_M_ values are decreased (Table 2, Fig. S3). The specificity constant for cephalothin hydrolysis was 10-fold lower than for wildtype KPC-2. However, the largest impact of the N170A substitution was on carbapenem hydrolysis. The N170A enzyme exhibited a 3000-fold reduction in *k*_cat_ and a 1000-fold decrease in *k*_cat_/*K*_M_ for meropenem hydrolysis (Fig. S2). In addition, with imipenem as the substrate, the N170A mutant displayed a 2200-fold reduction in *k*_cat_ as compared with wildtype KPC-2 and a decrease in *K*_M_ to below the measurable limits with an estimated *K*_M_ of ≤0.6 μM and *k*_cat_/*K*_M_ of ≥3 × 10^4^ M^−1^ s^−1^ (Table 2, Fig. S1).

**Figure 6:**
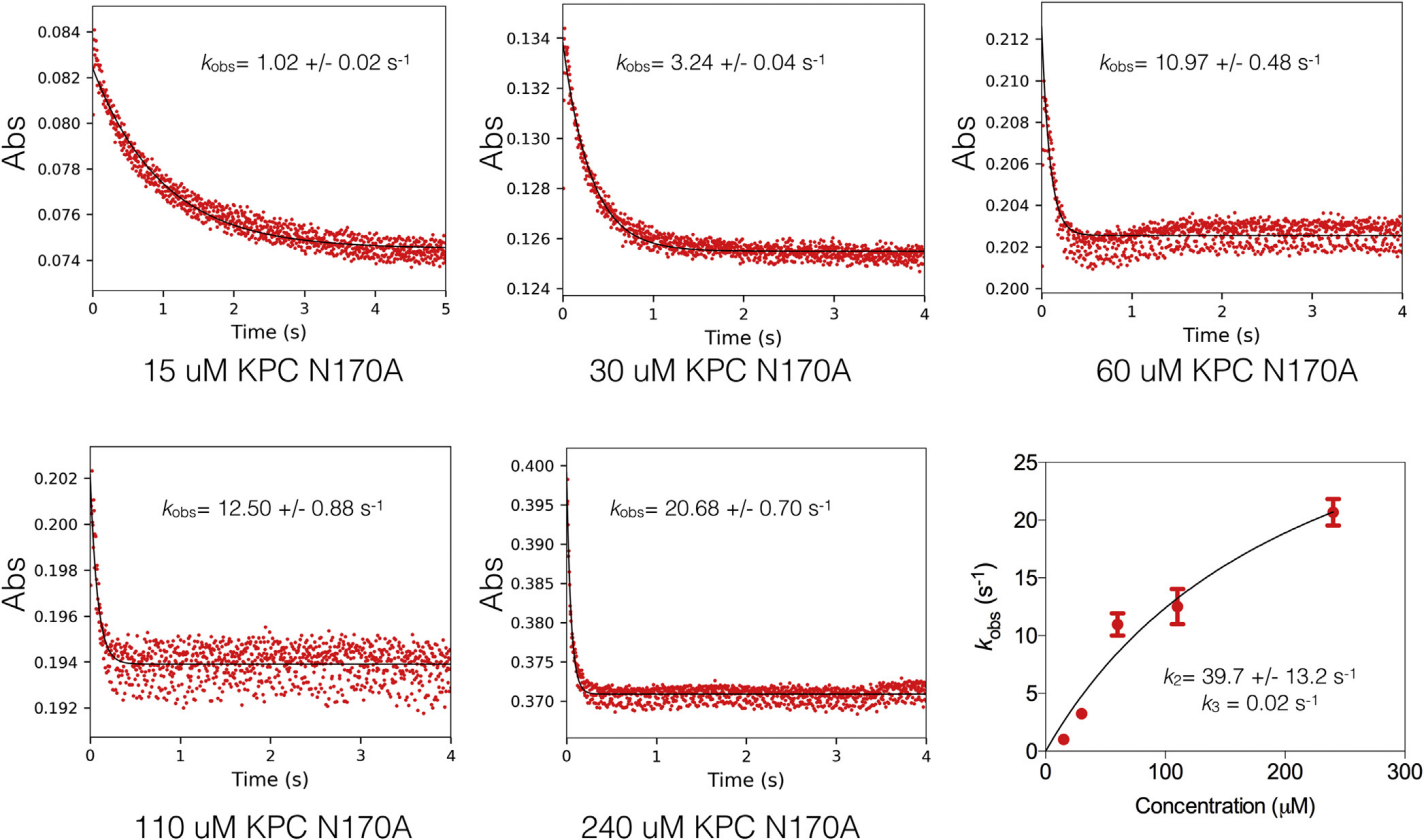
**Single turnover kinetic analysis of KPC-2 N170A hydrolysis of imipenem.** Imipenem10 μM, was used with increasing concentrations of KPC-2 enzyme as indicated below each plot. Details are as described for Figure 5.

Since sharply decreased values of both *k*_cat_ and *K*_M_ are consistent with impaired deacylation in the β-lactamase kinetic scheme (Fig. 2), we hypothesized that the N170A substitution results in a decreased rate of deacylation of the carbapenem substrates. To test this hypothesis, single turnover kinetics were performed for the N170A enzyme with imipenem, revealing a modest 5-fold decrease in the acylation rate (*k*_2_) but a 2800-fold decrease in the deacylation rate (*k*_3_) as compared with wildtype KPC-2 such that deacylation is strongly rate limiting for imipenem hydrolysis in the mutant (Table 3, Fig. 6).

We also noted in the N170A single turnover experiments that the amplitude of the reaction corresponded to less imipenem reacting (∼3 μM) than the substrate concentration used (10 μM) (Fig. 6). Further single turnover experiments with longer time frames indicated that the reaction is biphasic, with a very slow second phase (Fig. S5). Upon completion of the second phase the amplitude accounts for the 10 μM reaction with imipenem. The fitting of the *k*_obs_ values for the slow rates yielded rate constants <1 s^−1^. Because *k*_cat_ for imipenem hydrolysis is 0.02 s^−1^, we cannot distinguish from this experiment whether *k*_cat_ is due to the slow phase of acylation or a slow deacylation step. Therefore, we determined the deacylation rate directly using an enzyme reactivation method, which yielded a *k*_3_ value of 0.04 s^−1^, similar to the value of 0.02 s^−1^ determined using the *k*_2_ value determined from the fast portion of the single turnover reaction and *k*_cat_ (45) (Fig. S6). Taken together with the biphasic kinetics results for other mutants described below, we conclude that the fast portion of the single turnover reaction represents the *k*_2_ value relevant to the Michaelis–Menten kinetic parameters.

Biphasic kinetics have also been reported in single turnover experiments with carbapenems for the GES-2 and -5 carbapenemases (32, 36). To explain these findings, it was proposed that the enzyme may recognize different conformers of the substrate or, alternatively, that there are two different enzyme populations that are acylated at different rates (32, 36). In this regard, recent molecular dynamics studies indicating alternate conformations of KPC-2 exist in solution, one of which is not catalytically competent, are consistent with the fast and slow rates observed here (26). In addition, it is possible that there are two binding sites for carbapenems in the mutant enzyme active sites. Each of these rationales are possible but cannot be distinguished from the single turnover results.

The greatly reduced *K*_M_ value for imipenem (≥450-fold) could also be indicative of a change in binding affinity for the substrate. The fit of *k*_obs_ values yielded a *K*_D_ of 220 μM indicating the substitution increases the binding affinity for imipenem approximately fivefold (Table 3, Fig. 6). Therefore, the increased affinity for imipenem contributes but the large decrease in *K*_M_ for N170A for imipenem hydrolysis is largely due to a decreased deacylation rate, consistent with the equation for *K*_M_ in the β-lactamase kinetic scheme (Fig. 2).

The results indicate that the KPC-2 N170A enzyme has catalytic properties for carbapenem catalysis similar to the TEM-1 and SHV-1 enzymes with very low *k*_cat_ values due to the slow deacylation of carbapenems (6, 31). This is an interesting finding given that Asn170 is conserved between KPC-2, TEM-1, and SHV-1. Thus, the presence of Asn170 promotes carbapenem hydrolysis in the context of the KPC-2 active site but not in the context of the TEM-1 or SHV-1 active sites. This suggests that the positioning and/or dynamics of other residues differ between the enzymes and results in an altered contribution of Asn170 to carbapenem catalysis. In addition, the contribution of Asn170 to the catalysis of ampicillin hydrolysis also depends on the context of the active site. The N170A substitution does not alter *k*_cat_/*K*_M_ for KPC-2-mediated ampicillin hydrolysis but results in a 17-fold decrease in the specificity constant for the TEM-1 enzyme (44). Therefore, although Asn170 is a conserved position, active site context has a strong influence on its role in catalysis.

Analysis of the crystal structures of GES-1 (a non-carbapenemase) and GES-2 (a carbapenemase) that differ only by a single amino acid (Gly170 for GES-1 and Asn170 for GES-2) provides insight into the catalytic properties of KPC-2 N170A. The C6-hydroxyethyl group, a common structural feature of the carbapenems, plays a central role. In GES-1 the hydroxyethyl group occupies the position normally used to bind the catalytic water molecule required for deacylation. In GES-2, the binding site for the catalytic water is retained because the Asn170 side chain sterically forces the C6-hydroxyethyl group of imipenem into a hydrogen bonding interaction with Asn132 (32). Similarly, analysis of the SFC-1 carbapenemase cocrystal structure with meropenem shows that the C6-hydroxyethyl group is restrained away from the catalytic water by hydrogen bonding to Asn132 while Asn170 and Glu166 coordinate the catalytic water molecule (7). Based on this evidence, we speculate that the impaired deacylation of imipenem observed with the KPC-2 N170A enzyme is due to the C6-hydroxyethyl side chain occupying the cavity created by the substitution and displacing the catalytic water molecule.

Our results indicate a key role for Asn170 in carbapenem hydrolysis catalyzed by KPC-2. In KPC-2, the Asn170 coordinates the deacylation water and may also reposition the C6-hydroxyethyl group of the carbapenems. This is clearly not the exclusive reason KPC-2 is a carbapenemase, however, since TEM-1 and many other class A enzymes contain Asn170 and yet do not hydrolyze carbapenems. A possible reason that TEM-1, despite containing Asn170 to restrain the position of the hydroxyethyl group, does not hydrolyze carbapenems may lie in its narrower active site compared with KPC-2, which could prevent the positioning of the hydroxyethyl group for hydrogen bonding with Asn132 (30, 46).

### X-ray structures of KPC-2 N170A apoenzyme and ampicillin, imipenem product complexes

**Figure 7:**
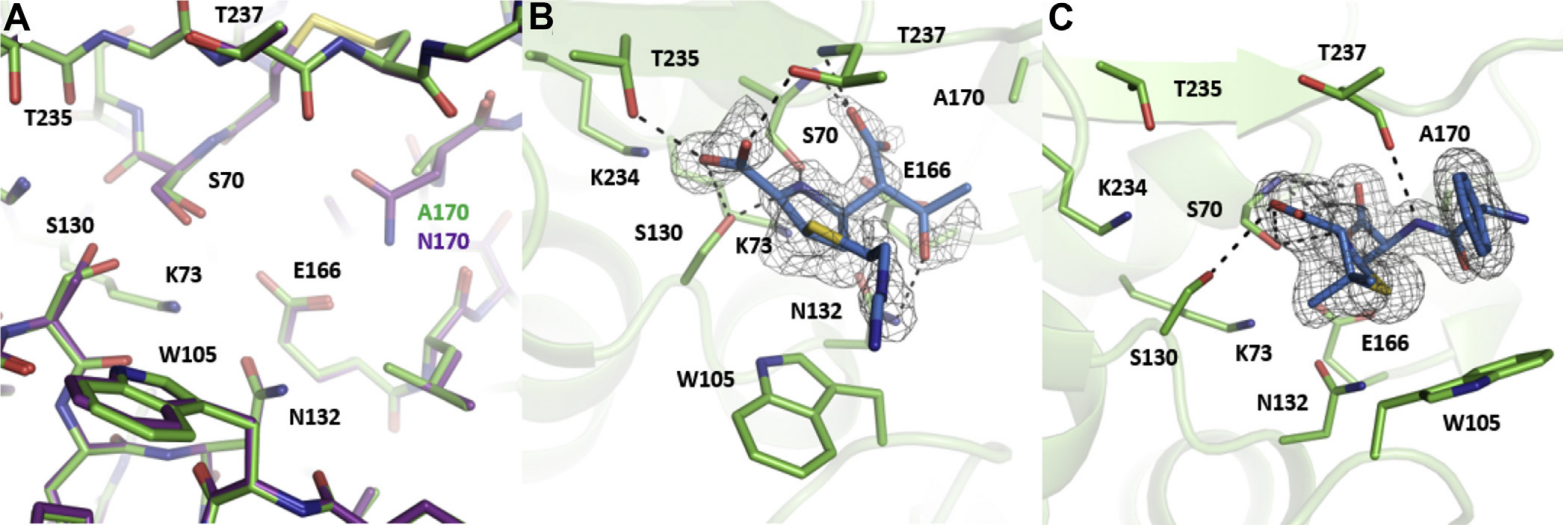
**Crystal structures of KPC-2 N170A enzyme.** The simulated annealing mFo – DFc composite omit maps are colored in *gray* and contoured at 2σ. *Black dashed lines* represent hydrogen bonds. *A*, superimposition of KPC-2 N170A (*green*, PDB code 6XD5) and KPC-2 wildtype (*purple*, PDB code 5UL8) apoenzymes. *B*, KPC-2 N170A in complex with hydrolyzed imipenem (PDB code 6XJ8). KPC-2 N170A residues are colored in *green*, and hydrolyzed imipenem is colored in *blue*. *C*, KPC-2 N170A in complex with hydrolyzed ampicillin (PDB code 6XD7). KPC-2 N170A residues are colored in *green*, and hydrolyzed ampicillin is colored in *blue*.

To further investigate the role of Asn170 in penicillin and carbapenem hydrolysis, we determined the X-ray structure of the KPC-2 N170A apoenzyme as well as product complexes with ampicillin and imipenem (Fig. 7). As seen in Figure 7*A*, the structure of the N170A apoenzyme is very similar to that of the wildtype KPC-2 apoenzyme with the only major difference being the smaller alanine side chain at position 170, which creates a pocket not found in wildtype and thus expands the volume of the active site in this region. Imipenem was soaked with a KPC-2 N170A crystal and the structure was determined, revealing a hydrolyzed product in the active site (Fig. 7*B*). Thus, despite the very slow deacylation rate of the N170A enzyme for imipenem, the reaction was completed in the crystals after soaking. The hydrolyzed imipenem makes similar interactions as those observed in the SFC-1 substrate and acyl-enzyme structures with meropenem (7). In all structures, the C3 carboxylate participates in hydrogen bonds with the Ser130, Thr235, and Thr237 side-chain hydroxyl groups. The placement of the newly generated carboxylate is similar to the recently determined imipenem product complex with OXA-48 (46). There appears to be a hydrogen bond between the product carboxylate group and the Glu166 side chain, likely due to the low pH of the ammonium sulfate crystallization conditions and the short soaking time during complex crystal preparation. The nitrogen from the hydrolyzed ring also makes hydrogen bonds with the Ser70 and Ser130 side-chain hydroxyl groups. The hydroxyl from the 6α-1R-hydroxyethyl side chain forms a hydrogen bond with Asn132 and is oriented away from the new carboxylate formed by ring hydrolysis. This orientation would not interfere with water activation and deacylation. However, the position of the hydroxyethyl group in the product complex may be different from that in the acyl-enzyme intermediate.

The structure of the ampicillin product complex with KPC-2 N170A shows differences with the imipenem product complex (Fig. 7*C*). The acylation and deacylation rates for ampicillin catalysis by the N170A enzyme are not known. However, since *k*_cat_ for ampicillin with the N170A enzyme is 600-fold higher than that for imipenem, deacylation of ampicillin must be at least 600-fold faster than imipenem (Table 2). The C3 carboxylate of the ampicillin product is not directly hydrogen bonded to Thr235 or Thr237 but retains hydrogen bonds to Ser70 and Ser130. In contrast to hydrolyzed imipenem, the two oxygens from the new carboxylate formed by hydrolysis each establishes a hydrogen bond with the backbone amide group of Ser70 in the oxyanion hole, while the hydrogen bond with Thr237 backbone is lost. The amide of ampicillin forms a hydrogen bond with the backbone oxygen atom of Thr237. The nitrogen of the dihydrothiazole ring also forms a hydrogen bond with Ser70. Furthermore, the benzyl ring of ampicillin is oriented to make a face-to-face interaction with the dihydrothiazole ring with extensive van der Waals interactions. This hydrophobic group of ampicillin also makes extensive van der Waals contacts with Trp105, which is shifted to an altered rotamer pointed down the active site. Taken together, the structures do not provide an obvious mechanism for why ampicillin is deacylated rapidly and imipenem slowly but do show the imipenem and ampicillin hydrolysis products both make extensive, but different, interactions with the active site.

### Role of Arg220 in β-lactam hydrolysis

In our structures of the KPC-2 apoenzyme and product complex with imipenem as well as published complexes of hydrolyzed faropenem with KPC-2 and meropenem with the S70A mutant of the SFC-1 carbapenemase, the guanidinium group of Arg220 makes hydrogen bonds with the Thr237 side chain to position it for interaction with the carbapenem C3 carboxylate group (7, 29, 30). In addition, it establishes an electrostatic environment favorable for the carbapenem C3 carboxylate group, which may contribute to substrate binding and catalysis (7, 30) (Fig. 4*E*). The steady-state kinetic parameters of the R220A and R220Q enzymes for ampicillin, cephalothin, imipenem, and meropenem hydrolysis are listed in Table 2 (Figs. S1–S4). Interestingly, both substitutions result in an ∼10-fold increase in *k*_cat_ for ampicillin catalysis, which, based on the β-lactamase mechanism, could be due to increases in the rate of acylation (*k*_2_) and/or deacylation (*k*_3_) (Fig. 2). *k*_cat_/*K*_M_ values for ampicillin hydrolysis are also increased approximately 10-fold for both substitutions. Since *k*_cat_/*K*_M_ reflects rates up to the formation of the acyl-enzyme, this result suggests that *k*_2_ is increased and/or the binding affinity of ampicillin is enhanced (*K*_D_). Taken together, however, the similar increases in both *k*_cat_ and *k*_cat_/*K*_M_ suggest that an increased acylation rate (*k*_2_) is probable. Thus, arginine at position 220 is not required and, in fact, is detrimental to ampicillin catalysis by KPC-2.

Similar to the observations with ampicillin, the R220A and R220Q substitutions result in increased *k*_cat_ values for cephalothin catalysis, indicating increased *k*_2_ and/or *k*_3_, while *k*_cat_/*K*_M_ values are similar to those of wildtype KPC-2 (Table 2) (Fig. S4). Thus, Arg220 does not contribute significantly to either ampicillin or cephalothin catalysis by KPC-2 and, in fact, limits *k*_cat_, since both substitutions result in higher *k*_cat_ values.

In contrast, the R220A and R220Q substitutions result in a modest decrease (∼2-fold) in *k*_cat_ values for hydrolysis of imipenem and a more severe (∼10-fold) decrease for meropenem, indicating decreased *k*_2_ and/or *k*_3_ values. *k*_cat_/*K*_M_ values, however, are higher than for wildtype KPC-2 for both imipenem and meropenem (Table 2, Fig. S1). Since *k*_cat_/*K*_M_ reflects rates up to the acyl-enzyme, this suggests an increased acylation rate (*k*_2_) and/or improved binding affinity for the carbapenems. Coupled with the fact that *k*_cat_ values are decreased, the results suggest an increased binding affinity for carbapenems due to substitution of Arg220. To test this hypothesis, single turnover kinetics were performed for the R220A and R220Q enzymes with imipenem as substrate, which indicated a 6-fold reduction in *k*_2_ for both mutants and a 2-fold reduction in *k*_3_ for R220A but a 2.4-fold increase in *k*_3_ for R220Q (Figs. S7 and S8). Thus, the largest effect of Arg220 substitutions is a decrease in the acylation rate (*k*_2_) so that acylation (R220Q) or neither step (R220A) becomes rate limiting in these mutants while deacylation limits the rate in wildtype KPC-2 as well as the N170A mutant. In addition, the fact that *k*_2_ is decreased and yet *k*_cat_/*K*_M_ is increased for imipenem catalysis suggests that the binding affinity is improved in the R220A and R220Q mutants. The *K*_D_ value, determined from the fit of *k*_obs_ values *versus* enzyme concentration (Figs. S7 and S8), indicates that the affinity of the R220A (188 μM) and R220Q (35 μM) mutants for imipenem is approximately 7-fold and 35-fold tighter, respectively, than that for wildtype KPC-2 (1240 μM) (Table 3). Although Arg220 positions the side chain of Thr237 to create a favorable electrostatic environment for the C3 carboxylate of carbapenems, removal of the positive charge in the R220A and R220Q mutants increases enzyme affinity for imipenem. However, the bound imipenem substrate is acylated approximately sixfold slower than in wildtype KPC-2 by the R220A and R220Q enzymes. This is consistent with the idea that a tighter ground state binding can lead to a higher energy barrier to reach the transition state for acylation, resulting in a reduced rate (39). Thus, the Arg220 residue functions to destabilize the bound carbapenem and increase the acylation rate.

Similar to the results with the N170A enzyme, the single turnover experiments with the R220A and R220Q enzymes generated progress curves that were biphasic and were fit with a double exponential function (Experimental Procedures, Figs. S7 and S8). However, in contrast to the N170A single turnover results, both phases of the reaction are apparent in the short timescale experiments (Figs. S7 and S8). The fast phase of the reaction generated acylation rates of 35 and 34 s^−1^ for R220A and R220Q, respectively (Figs. S7 and S8). The fitting of the *k*_obs_ values for the slow rates yielded rate constants <1 s^−1^. *k*_cat_ values for imipenem hydrolysis for R220A and R220Q, however, are 13 and 27 s^−1^, respectively (Table 2, Fig. S1). Thus, the acylation rate constants generated from the slow phase of the reaction would be strongly rate limiting and are not consistent with the observed *k*_cat_ values. In contrast, the *k*_2_ value from the fast phase is consistent with the observed Michaelis–Menten kinetic parameters. Therefore, the *k*_2_ fast rate constant is reported in Table 3 and was used for the discussion above.

### Role of Thr237 in β-lactam hydrolysis

The Thr237 residue forms a hydrogen bond with the C3 carboxylate of meropenem in the structure of the complex with the carbapenemase, SFC-1 S70A, and in the complex of hydrolyzed faropenem with KPC-2 (7, 30). Therefore, Thr237 may enhance substrate binding affinity and catalysis by interacting with C3/C4 carboxylate that is found in penicillins, cephalosporins, and carbapenems. As noted above, Thr237 also forms hydrogen bonds with Arg220, which may impact the positioning of the Thr237 hydroxyl group. Interestingly, the T237A substitution has similar effects as the R220A and R220Q substitutions on kinetic parameters for β-lactam hydrolysis. Similar to the Arg220 mutants, the T237A enzyme exhibits increased *k*_cat_ and decreased *K*_M_ values for ampicillin hydrolysis, resulting in a 40-fold increase in *k*_cat_/*K*_M_ compared with wildtype KPC-2. The T237A substitution also results in a modest decrease in the *k*_cat_ for imipenem and a more drastic decrease in *k*_cat_ for meropenem hydrolysis, suggesting decreased *k*_2_ and/or *k*_3_ values (Table 2, Figs. S1 and S2). In the case of imipenem, *k*_cat_/*K*_M_ is modestly increased relative to wildtype KPC-2, whereas for meropenem, *k*_cat_/*K*_M_ is similar to that for wildtype KPC-2. Since *k*_cat_/*K*_M_ reflects rates up to the acyl-enzyme, the results suggest an increased acylation rate (*k*_2_) and/or improved binding affinity for imipenem. Coupled with the fact that *k*_cat_ values are decreased, the results suggest an increased binding affinity for imipenem due to the T237A substitution.

Single turnover kinetics was performed with the T237A enzyme with imipenem to determine the acylation (*k*_2_) and, by inference, the deacylation (*k*_3_) rate. As with the Arg220 mutants, single turnover experiments with the T237A enzyme and imipenem resulted in biphasic kinetics (Fig. S9). Similar to the Arg220 mutants, the *k*_2_ value determined from the slow phase of the reaction (0.07 s^−1^) would be strongly rate limiting and is not consistent with the observed *k*_cat_ value of 8.9 s^−1^ determined in steady-state measurements (Table 2). It is noteworthy that the Arg220 and Thr237 residues, which both exhibit biphasic kinetics when mutated, are in the same region of the active site and are both thought to contribute to substrate binding through interactions with the C3 carboxylate group. Changes in this region of the structure could also affect the protonation of the C2 position of imipenem. Protonation of C2 would facilitate tautomerization of imipenem to the Δ1-pyrroline form that could exhibit altered acylation kinetics (25) (Fig. 3). Thus, changes in substrate binding properties or the imipenem protonation state might also explain the observed biphasic kinetics.

The results for the fast phase of the acylation reaction are consistent with the Michaelis–Menten kinetic parameters and are very similar to those obtained for the R220A enzyme with imipenem. A 12-fold reduction in *k*_2_ and 2.5-fold decrease in *k*_3_ were observed (Table 3). Thus, the largest effect of the T237A substitution is a decrease in the acylation rate (*k*_2_) such that, like the Arg220 mutants, deacylation of the T237A enzyme is not rate limiting. In addition, the fact that *k*_2_ is decreased and yet *k*_cat_/*K*_M_ is increased for imipenem catalysis suggests that binding affinity is improved in the T237A enzyme. The fitting of *k*_obs_ to enzyme concentration in the single turnover experiment indicates that the affinity of the T237A enzyme for imipenem (24 μM) is increased 50-fold compared with wildtype KPC-2. Thus, the acylation and deacylation rates are decreased but the large increase in affinity for imipenem results in the specificity constant of the T237A mutant being higher than that of wildtype KPC-2. Therefore, although Thr237 is positioned to make a hydrogen bond with the C3 carboxylate of carbapenems, removal of the hydrogen bonding potential in the T237A mutant actually increases enzyme affinity for imipenem. Paradoxically, Arg220 and Thr237, which are positioned to interact with the imipenem C3 carboxylate group, are detrimental to the affinity of the enzyme for imipenem but are important for subsequent acylation of the bound substrate. As noted above, an increased binding affinity for the substrate could lead to a decreased reaction rate (*k*_2_) owing to the higher energy barrier to the acylation transition state from the tightly bound substrate (39). Alternatively, imipenem may bind more tightly to the mutant enzymes, but in an altered position in the active site relative to catalytic residues such as Ser70 and Lys73, thereby leading to a slower reaction.

### Role of Thr235 in β-lactam hydrolysis

A serine or threonine residue is strongly conserved at position 235 in class A β-lactamases, and X-ray structures in complex with β-lactam substrates, including carbapenems, show that the hydroxyl group makes a hydrogen bond to the C3/C4 carboxylate group (7, 30, 40, 47, 48, 49). Consistent with an important role for Thr235 in substrate binding, it was not possible to determine *k*_cat_ and *K*_M_ for the T235A enzyme with ampicillin or cephalothin hydrolysis because of a very high *K*_M_ (Table 2) (Figs. S3 and S4). *k*_cat_/*K*_M_ values were determined at substrate concentrations <<*K*_M_ and revealed a sevenfold increase relative to wildtype KPC-2 for ampicillin hydrolysis. Since *K*_M_ is much higher than the wildtype value, this indicates that *k*_cat_ is also increased for ampicillin hydrolysis and therefore *k*_2_ and/or *k*_3_ is increased for the T235A enzyme. This is similar to the observations with the R220A/Q and T237A mutants where the ampicillin *k*_cat_ was increased upon substitution and suggests that the threonine at position 235 actually restricts ampicillin turnover compared with alanine at position 235. It was also not possible to determine *k*_cat_ and *K*_M_ for cephalothin hydrolysis by the T235A enzyme due to a very high *K*_M_ value. *k*_cat_/*K*_M_ was determined at substrate concentrations <<*K*_M_ and was found to be 20-fold lower than for the wildtype for cephalothin hydrolysis (Table 2) (Fig. S4). Therefore, with cephalothin it is not possible to make inferences about the relative change of *k*_cat_ compared with wild type.

It was possible to determine *k*_cat_ and *K*_M_ values for the T235A enzyme for carbapenem hydrolysis, and *k*_cat_ was reduced >10-fold for both imipenem and meropenem, indicating *k*_2_ and/or *k*_3_ are reduced for carbapenem hydrolysis (Table 2, Figs. S1 and S2). *k*_cat_/*K*_M_ values were also reduced approximately 10-fold for both imipenem and meropenem, suggesting that the acylation rate (*k*_2_) and/or affinity (*K*_D_) is reduced for carbapenems. This is in contrast to the results for the R220A/Q and S237A mutants with imipenem where *k*_cat_/*K*_M_ was increased. Despite Thr235 also being a C3 carboxylate binding residue, it exhibits altered hydrolysis kinetics compared with these mutants.

We were not able to accurately measure *k*_2_ and *K*_D_ values for imipenem catalysis by the T235A enzyme. However, *k*_obs_ values in single-turnover experiments were >100 s^−1^ at high T235A enzyme concentrations, suggesting that deacylation is rate limiting and therefore that Thr235 contributes strongly to the deacylation reaction (Fig. S10). Loss of the C3 carboxylate binding interaction with Thr235 might be expected to reduce both the acylation and deacylation rates since it is an anchor to hold the carbapenem in position for catalysis. The precise impact on acylation is unclear, although the acylation rate is >100 s^−1^ based on *k*_obs_ values at high enzyme concentrations, suggesting a modest effect of the substitution on the acylation rate (Fig. S10). Therefore, Thr235 contributes disproportionally to the deacylation reaction, suggesting that the interaction is critical for positioning the acyl-enzyme for attack of the hydrolytic water.

## Conclusions

The inhibitory effect of carbapenem antibiotics for class A β-lactamases has been attributed to multiple possibilities such as tautomerism of the substrate, inactivation of the deacylating water molecule, and displacement of the β-lactam carbonyl from the oxyanion hole (6, 10, 25, 26, 43, 46). The carbapenemase activity of KPC-2 stems from the enzyme’s ability to overcome each of these inhibitory traps and significantly increase the deacylation rate of carbapenem turnover. Our results indicate that multiple residues contribute to increased carbapenem hydrolysis by KPC-2.

The N170A substitution results in a drastic decrease in *k*_cat_ for imipenem hydrolysis due to a large reduction in the deacylation rate. The Asn170 side chain may facilitate deacylation by restraining the movement of the hydroxyethyl group to block it from interacting with the catalytic water molecule. Since Asn170 is conserved in non-carbapenemases such as TEM-1, these results indicate that the enzyme context is important for the contribution of Asn170 to carbapenem hydrolysis. The larger size and increased hydrophobicity of the KPC-2 active site compared with non-carbapenemase class A enzymes may explain why Asn170 promotes deacylation in KPC-2 but not other class A enzymes (30).

The Arg220 and Thr237 residues play an important role in the acylation reaction for carbapenem substrates. Surprisingly, substitution of these residues with alanine improves the binding affinity for carbapenems but decreases the acylation rate (*k*_2_) (Tables 2 and 3). Thus, the role of these residues may be ground state destabilization of the enzyme–substrate complex to lower the energy needed to reach the acylation transition state. In contrast, alanine substitutions at Arg220 and Thr237 result in sharply increased *k*_cat_ values for ampicillin hydrolysis. The *k*_2_ and *k*_3_ values for ampicillin catalysis by wildtype KPC-2 and the mutants are not known but Arg220 and Thr237 clearly have different roles in ampicillin *versus* carbapenem hydrolysis.

It is paradoxical that alanine mutants of the Arg220 and Thr237 residues, which are positioned to facilitate interaction with the substrate C3 carboxylate, exhibit increased affinity for carbapenems. One possibility is that the elimination of Arg220 and/or Thr237 interactions results in enhanced interactions of the C3 carboxylate with other suitably positioned residues including Ser130, Lys234, and Thr235 that more than compensates for the loss of the Arg220 or Thr237 interaction.

The results of this study suggest that several KPC-2 active site residues work in concert to achieve carbapenem hydrolysis. This, in turn, suggests that KPC-mediated hydrolysis may be sensitive to modifications of the carbapenem scaffold to create suboptimal interactions with the KPC-2 active site while retaining the ability to inhibit penicillin binding proteins. Of particular interest would be modifications that alter interactions with Asn170 or changes to the C3 carboxylate that enhance interactions with Arg220 and Thr237. Changes to the 6α-1R-hydroxyethyl group could disrupt Asn170 function and impair catalysis while altering the charge or position of the C3 group such as replacement with sulfonic acid may lead to tighter binding of the ground state or altered positioning of substrate or acyl-enzyme intermediate with respect to key catalytic residues such as Lys73 or Glu166.

## Experimental procedures

### Bacterial strains and plasmids

*E. coli* K12 XL1-Blue strain (*rec*A1 *end*A1 *gyr*A96 *thi*-1 *hsd*R17 *sup*E44 *rel*A1 *lac* [F’ *proAB lacI*^*q*^*ZΔM15 Tn*10 (Tet^r^)]) was obtained from Stratagene (La Jolla, CA, USA) and used for site-directed mutagenesis experiments. The *E. coli* RB791 strain was used for protein expression and purification (50). As previously described, the *bla*_KPC-2_ gene was inserted in the pTP123 plasmid (51, 52). Subsequently, the *bla*_KPC-2_-pTP123 construct was used as a template for site-directed mutagenesis and expression of wildtype and mutant enzymes in *E. coli* RB791.

### Site-directed mutagenesis

The KPC-2 variants including E166A, N170A, R220A, R220Q, T235A, and T237A were generated by the Quik-Change method utilizing a single primer to incorporate each mutation. The thermostable proofreading polymerase enzyme, *Pfu*, was used for the PCR reactions. The primers were purchased from Integrated DNA Technologies (Coralville, IA, USA). The following is the list of primers used to introduce mutations (underlined) into pTP123 KPC-2:

E166A: 5′-GGACCGCTGGGCCCTGGAGCTGAAC-3′

N170A: 5′-GGGAGCTGGAGCTGGCCTCCGCCAT-3′

R220A: 5′-CCGGCAACCACGCGATCCGCGCGGC-3′

R220Q: 5′-CCGGCAACCACCAAATCCGCGCGGC-3′

T235A: 5′-GCAGTCGGAGACAAGGCCGGAACCT-3′

T237A: 5′-GGAGACAAAACCGGGGCCCTGCGGAG-3′

The PCR product was digested with *Dpn*I enzyme for 8 h at 37 °C and was then used for transformation of competent *E. coli* XL1-Blue cells. All mutant constructs were confirmed by DNA sequencing (Lonestar Labs, Houston, TX, USA).

### Protein purification

The KPC-2 β-lactamase and its mutants were purified as described previously (51). Briefly, the periplasmic fraction was bound to a HiTrap SP column by adjusting the pH to 6.0 using MES acid. The bound protein was eluted using an NaCl gradient. Subsequently, the β-lactamase-containing fractions were subjected to gel filtration chromatography and the purity of the β-lactamase enzyme was assessed using SDS-PAGE. Samples with sufficient purity were pooled and concentrated. The protein concentrations were determined by measuring their absorbance at 280 nm, using the extinction coefficient 39,545 M^−1^ cm^−1^ as calculated by the “ProtParam” tool from the Swiss Institute of Bioinformatics online resource portal (53).

### X-ray crystallography

Apo KPC-2 N170A crystals were grown in 2 M ammonium sulfate and 5% (v/v) ethanol with microseeding. KPC-2 N170A hydrolyzed β-lactam complexes were obtained by soaking apo crystals in solutions containing 1.44 M sodium citrate/20 mM ampicillin and 1.44 M sodium citrate/20 mM imipenem for 5 min before cryoprotection with 1.44 M sodium citrate and 20% (v/v) glycerol and flash cooling in liquid nitrogen. X-ray diffraction data were collected at the Advanced Photon Source beamline 22-ID. X-ray diffraction data were indexed and integrated with iMosflm (54) and scaled with Aimless (55) from the CCP4 suite (56). Phases were obtained with Phaser (57) from PHENIX software suite (58) with the KPC-2 model (Protein Data Bank [PDB] code 5UL8). Structure refinements were performed with phenix.refine (59) of the PHENIX software suite and WinCoot (60) was used for model building. Simulated annealing mFo – DFc composite omit maps were created using the composite omit map program (61) from the PHENIX software suite. Ligand geometry restrain information was obtained using the program eLBOW (62) from the PHENIX software suite. The final model qualities were assessed with MolProbity (63). All protein structure figures were generated using PyMOL 1.3 (Schrödinger). Statistics for data collection and refinement are found in Table S1.

### Determination of steady state kinetic parameters

Michaelis–Menten kinetic parameters for KPC-2 and the variant enzyme–substrate pairs were determined at 25 °C in 50 mM sodium phosphate buffer, pH 7.0, containing 0.1 mg/ml BSA. In the case of ampicillin as substrate, BSA was omitted from the assay owing to overlap in absorbance with the substrate. The concentration of enzyme varied depending on the enzyme–substrate pair. Initial velocities of β-lactam hydrolysis were measured on a Beckman-Coulter spectrophotometer model DU-800 (Fullerton, CA, USA) using the following extinction coefficients: imipenem, Δε_295_ = −9000 M^−1^ cm^−1^; meropenem, Δε_295_ = −10,940 M^−1^ cm^−1^; ceftazidime Δε_295_ = −7600 M^−1^ cm^−1^; ampicillin, Δε_235_ = −900 M^−1^ cm^−1^. GraphPad Prism 8 was used to obtain the steady-state parameters by nonlinear least squares fit of the data to the Michaelis–Menten equation *v = k*_cat_[S]/(*K*_m_ + [S]). When the *V*_max_ could not be achieved due to a high *K*_m_, the second-order rate constant at steady state, *k*_cat_*/K*_m_, was determined by fitting the progress curves to the equation *v = k*_cat_/*K*_m_[E][S], where [S] << *K*_m_ (eq. 1). A total of 2 to 5 determinations of initial velocities were done for each substrate concentration and enzyme. The error for *k*_cat_ and *K*_m_ is standard error of the fit of the initial velocity determinations to the Michaelis–Menten equation. *k*_cat_/*K*_m_ was determined by dividing the *k*_cat_ by the *K*_m_ value. The error on *k*_cat_/*K*_m_ was determined by propagating the error of both *k*_cat_ and *K*_m_ by calculating the square root of the sum of the fractional errors of *k*_cat_ and *K*_m_ squared (64).

### Determination of microscopic rate constants describing acylation and deacylation

The acylation rate (*k*_2_) for imipenem hydrolysis by KPC-2 and the mutant enzymes was measured by single turnover transient kinetic analysis using a KinTek Stopped-Flow instrument. The reaction was initiated using a low concentration of imipenem (10 μM, Δε_295_ = −9000 M^−1^ cm^−1^) and excess enzyme to enforce single turnover conditions. The absorbance *versus* time data were fit to Equation 2, and the enzyme concentration was increased until the acylation rate was saturated. The data were fit to Equation 2. For some KPC-2 mutants, biphasic kinetics were observed and the resulting progress curves were fit to Equation 3.

(2) $$A_{t}=\;{A_{1}}^{\left(-k_{obs}t\right)}\;+\;c$$

(3) $$A_{t}=\;{A_{1}}^{\left(-k_{obs-f}t\right)}\;+\;{A_{2}}^{\left(-k_{obs-s}t\right)}\;+\;c$$

For these equations, *A*_t_ is the absorbance at time *t*, *A*_1_ and *A*_2_ are the absorbance amplitudes describing the fast and slow phases, and *k*_obs_ is the apparent rate constant for the reaction. For Equation 3, *k*_obs-f_ is the observed rate constant for the fast phase and *k*_obs-s_ is the observed rate constant for the slow phase. The observed rate constants were plotted against the respective enzyme concentration and fit to a hyperbola (Equation 4) where E_0_ is enzyme concentration and *K*’ is (*k*_−1_ + *k*_2_)/*k*_1_ (or *k*_−1_/*k*_1_ when *k*_−1_ >> *k*_2_) to determine the acylation rate constant (*k*_2_) (32, 37, 38, 65). The deacylation rate constant (*k*_3_) was calculated from *k*_cat_ (determined from steady-state kinetics), using Equation 5 (35).

(4) $$k_{obs}\;=\;\frac{k_{2}\left[E_{0}\right]}{K^{\prime }\;+\;\left[E_{0}\right]}$$

(5) $$k_{cat}\;=\;\frac{k_{2}\;.\;k_{3}}{k_{2}\;+\;k_{3}}$$

The deacylation rate constant (*k*_3_) for the N170A mutant was also determined using enzyme reactivation kinetics (45). A total of 100 nM of N170A enzyme was incubated with imipenem at a concentration of at least 10-fold the *K*_m_ value for 60 s. To observe the release of imipenem from the active site, the reaction mixture was diluted 100-fold into 500 μM (>5-fold *K*_M_) of the reporter substrate nitrocefin. The hydrolysis of nitrocefin was monitored at 482 nm to observe recovery of the enzyme. The observed reaction was fit to Equation 6 where A_0_ represents the initial absorbance and A_t_ represents the absorbance at time t, and *v*_s_ is the steady-state velocity of nitrocefin hydrolysis and *k*_3_ is the recovery of enzymatic activity, representing deacylation.

(6) $$A_{t}\;=\;A_{0}\;+\;v_{s}t\;-\;\frac{v_{s}}{k_{3}}\left(1-e^{-k_{3}t}\right)$$

## Data availability

Coordinates and structure factors have been deposited in the PDB under accession codes 6XD5 (KPC-2 N170 apo), 6XJ8 (KPC-2 N170A/hydrolyzed imipenem), and 6XD7 (KPC-2 N170A/hydrolyzed ampicillin). Other data are available upon request from the corresponding author.

## Conflict of interest

The authors declare that they have no conflicts of interest with the contents of this article.
