# Supplementary material for: KPC-2 β-lactamase enables carbapenem antibiotic resistance through fast deacylation of the covalent intermediate
Source: J Biol Chem. 2020 Dec 10;296:100155. doi: 10.1074/jbc.RA120.015050 (PMC7895804; doi:10.1074/jbc.RA120.015050)
Supplement: Supplementary Figures and Tables [file mmc1.docx]

**Supporting Information**

KPC-2 β-lactamase Enable Carbapenem Antibiotic Resistance Through Fast Deacylation of the Covalent Intermediate

Shrenik C. Mehta^1^, Ian M. Furey^1^, Orville A. Pemberton^2^, David M. Boragine^3^, Yu Chen^2^ and Timothy Palzkill^1*^

**Supplemental Table 1.** Statistics for X-ray structure determinations.

**Supplemental Figure 1.** Determination of Michaelis-Menten kinetic parameters for imipenem hydrolysis for KPC-2 and mutants.

**Supplemental Figure 2.** Determination of Michaelis-Menten kinetic parameters for meropenem hydrolysis for KPC-2 and mutants.

**Supplemental Figure 3.** Determination of Michaelis-Menten kinetic parameters for ampicillin hydrolysis for KPC-2 and mutants.

**Supplemental Figure 4.** Determination of Michaelis-Menten kinetic parameters for cephalothin hydrolysis for KPC-2 and mutants.

**Supplemental Figure 5.** Single turnover kinetic analysis of KPC-2 N170A enzyme hydrolysis of imipenem using different timescales.

**Supplemental Figure 6.** Determination of KPC-2 N170A deacylation rate (*k*_3_) using enzyme reactivation kinetics.

**Supplemental Figure 7.** Single turnover kinetic analysis of KPC-2 R220A enzyme hydrolysis of imipenem.

**Supplemental Figure 8.** Single turnover kinetic analysis of KPC-2 R220Q enzyme hydrolysis of imipenem.

**Supplemental Figure 9.** Single turnover kinetic analysis of KPC-2 T237A enzyme hydrolysis of imipenem.

**Supplemental Figure 10.** Single turnover kinetic analysis of KPC-2 T235A enzyme hydrolysis of imipenem.

Table S1. X-ray crystallographic statistics for KPC-2 N170A apo and complexes of hydrolyzed imipenem and ampicillin

|  | **KPC-2 N170A Apo** | **KPC-2 N170A Hydrolyzed Imipenem** | **KPC-2 N170A Hydrolyzed Ampicillin** |
| --- | --- | --- | --- |
| PDB code | 6XD5 | 6XJ8 | 6XD7 |
| Data collection |  |  |  |
| Space group | P 2 2_1_ 2_1_ | P 2 2_1_ 2_1_ | P 2 2_1_ 2_1_ |
| Cell dimensions |  |  |  |
| *a*, *b*, *c* (Å) | 56.26, 60.05, 77.99 | 56.60, 59.36, 77.62 | 56.83, 59.38, 77.31 |
| a, b, c (°) | 90.00, 90.00, 90.00 | 90.00, 90.00, 90.00 | 90.00, 90.00, 90.00 |
| Wavelength (Å) | 1.00 | 1.00 | 1.00 |
| Resolution (Å) | 36.33–1.20 (1.24–1.20) | 36.23–2.05 (2.12–2.05) | 32.40–1.65 (1.71–1.65) |
| No. of unique reflections | 82,727 (7,811) | 16,583 (1,638) | 31,944 (3,137) |
| *<I>*/σ<*I>* | 6.9 (1.5) | 5.1 (3.1) | 7.4 (1.6) |
| Completeness (%) | 99.4 (94.8) | 97.7 (99.3) | 99.3 (99.2) |
| Redundancy | 6.7 (4.8) | 6.4 (6.9) | 6.6 (6.9) |
| *R*merge | 0.161 (1.487) | 0.226 (0.482) | 0.148 (1.870) |
| CC1/2 | 0.99 (0.35) | 0.96 (0.70) | 0.99 (0.39) |
| Reﬁnement |  |  |  |
| Resolution (Å) | 36.33–1.20 (1.24–1.20) | 36.23–2.05 (2.12–2.05) | 32.40–1.65 (1.71–1.65) |
| No. of reﬂections | 82,672 (7,766) | 16,583 (1,638) | 31,931 (3,131) |
| *R*work/*R*free | 0.1552/0.1786 | 0.1858/0.2363 | 0.1570/0.2037 |
| No. of non-hydrogen atoms | 2,401 | 2154 | 2,163 |
| Protein | 2,048 | 1983 | 1,983 |
| Ligands | 26 | 21 | 31 |
| Solvent | 327 | 150 | 149 |
| B-factors (Å^2^) |  |  |  |
| Protein | 14.83 | 24.78 | 29.52 |
| Ligands | 30.11 | 35.94 | 37.07 |
| Solvent | 36.85 | 32.05 | 42.73 |
| RMSD |  |  |  |
| Bond length (Å) | 0.005 | 0.007 | 0.006 |
| Bond angle (°) | 0.90 | 0.91 | 0.91 |
| Ramachandran |  |  |  |
| Favored (%) | 98.50 | 96.96 | 98.86 |
| Allowed (%) | 1.50 | 3.04 | 1.14 |
| Outliers (%) | 0.00 | 0.00 | 0.00 |

*Values in parentheses are for highest-resolution shell.

Figure S1

**Figure S1**. Determination of Michaelis-Menten kinetic parameters for imipenem hydrolysis for KPC-2 and mutants. Initial velocities normalized to enzyme concentration are shown in red and plotted on the Y-axis versus imipenem concentration on the X-axis. Each initial velocity measurement was repeated at least twice and the error bars on the points indicate the standard error of the mean. The data was fit to the Michaelis-Menten equation to determine *k*_cat_, *K*_M_ and *k*_cat_/*K*_M_. Enzyme concentrations used were wt KPC-2, 100 nM; 10 nM N170A, 1 μM; R220A; R220Q, 5 nM; T235A, 100 nM; T237A, 100 nM.

Figure S2

**Figure S2**. Determination of Michaelis-Menten kinetic parameters for meropenem hydrolysis for KPC-2 and mutants. Initial velocities normalized to enzyme concentration are shown in red and plotted on the Y-axis versus imipenem concentration on the X-axis. The data was fit to the Michaelis-Menten equation to determine *k*_cat_, *K*_M_ and *k*_cat_/*K*_M_. Enzyme concentrations used were wt KPC-2, 100 nM; N170A 1 μM; R220A, 500 nM; R220Q, 500 nM; T235A, 500 nM; T237A, 500 nM.

Figure S3

**Figure S3**. Determination of Michaelis-Menten kinetic parameters for ampicillin hydrolysis for KPC-2 and mutants. Initial velocities normalized to enzyme concentration are shown in red and plotted on the Y-axis versus imipenem concentration on the X-axis. The data was fit do the Michaelis-Menten equation to determine *k*_cat_, *K*_M_ and *k*_cat_/*K*_M_. Enzyme concentrations used were wt KPC-2, 100 nM; N170A, 50 nM; R220A, 20 nM; R220Q, 10 nM; T235A, 5 nM; T237A, 5 nM.

Figure S4

**Figure S4**. Determination of Michaelis-Menten kinetic parameters for cephalothin hydrolysis for KPC-2 and mutants. Initial velocities normalized to enzyme concentration are shown in red and plotted on the Y-axis versus imipenem concentration on the X-axis. The data was fit do the Michaelis-Menten equation to determine *k*_cat_, *K*_M_ and *k*_cat_/*K*_M_. Enzyme concentrations used were wt KPC-2, 10 nM; N170A, 100 nM; R220A, 50 nM; R220Q, 10 nM; T235A, 1 μM; T237A, 10 nM.

Figure S5

**Figure S5**. Single turnover kinetic analysis of KPC-2 N170A enzyme hydrolysis of imipenem using different timescales. 1 μM of imipenem was used with increasing concentrations of N170A enzyme as indicated below each plot. Absorbance is shown on the Y-axis and time in seconds on the X-axis. The left panel shows the fast phase of the reaction while the right panel shows the slow phase. Note that the left and right panels are from the same data set but viewed at different timescales. The *k*_obs_ value obtained from fitting an exponential equation is indicated for each plot. **A**. 1 uM imipenem, 5 uM KPC-2 N170A enzyme. **B**. 1 uM imipenem, 25 uM KPC-2 N170A enzyme. **C**. 1 uM imipenem, 37.5 uM KPC-2 N170A enzyme.

Figure S6

**Figure S6**. Determination of KPC-2 N170A deacylation rate (*k*_3_) using enzyme reactivation kinetics. The N170A enzyme was incubated with imipenem at a concentration of at least 10-fold the *K*_m_ value. To observe the release of imipenem from the active-site, the reaction mixture was diluted 100-fold into of the reporter substrate nitrocefin. The hydrolysis of nitrocefin was monitored at 482nm to observe recovery of the enzyme (Materials and Methods).

Figure S7. R220A-imipenem

**Figure S7**. Single turnover kinetic analysis of KPC-2 R220A enzyme hydrolysis of imipenem. 10 μM of imipenem was used with increasing concentrations of KPC-2 enzyme as indicated below each plot. Absorbance is shown on the Y-axis and time in seconds on the X-axis. The *k*_obs_ value obtained from fitting a double exponential equation is indicated for each plot. At bottom right is the fit of the *k*_obs_ values versus the KPC-2 enzyme concentrations to a hyperbola to obtain the acylation rate (*k*_2_).

Figure S8. R220Q- imipenem

**Figure S8**. Single turnover kinetic analysis of KPC-2 R220Q enzyme hydrolysis of imipenem. 10 μM of imipenem was used with increasing concentrations of KPC-2 enzyme as indicated below each plot. Absorbance is shown on the Y-axis and time in seconds on the X-axis. The *k*_obs_ value obtained from fitting a double exponential equation is indicated for each plot. At bottom right is the fit of the *k*_obs_ values versus the KPC-2 enzyme concentrations to a hyperbola to obtain the acylation rate (*k*_2_).

Figure S9. T237A-imipenem

**Figure S9**. Single turnover kinetic analysis of KPC-2 T237A enzyme hydrolysis of imipenem. 10 μM of imipenem was used with increasing concentrations of T237A enzyme as indicated below each plot. Absorbance is shown on the Y-axis and time in seconds on the X-axis. The *k*_obs_ value obtained from fitting a double exponential equation is indicated for each plot. At bottom right is the fit of the *k*_obs_ values versus the KPC-2 enzyme concentrations to a hyperbola to obtain the acylation rate (*k*_2_).

Figure S10. T235A-imipenem

**Figure S10**. Single turnover kinetic analysis of KPC-2 T235A enzyme hydrolysis of imipenem. 10 μM of imipenem was used with increasing concentrations of T235A enzyme as indicated below each plot. Absorbance is shown on the Y-axis and time in seconds on the X-axis. The *k*_obs_ value obtained from fitting a double exponential equation is indicated for each plot.
